# Practical Considerations of Wastewater–Seawater Integrated Reverse Osmosis: Design Constraint by Boron Removal

**DOI:** 10.3390/membranes11040240

**Published:** 2021-03-28

**Authors:** Chulmin Lee, Yesol Kang, Dong-Ho Kim, In S. Kim

**Affiliations:** Global Desalination Research Center, School of Earth Sciences and Environmental Engineering, Gwangju Institute of Science and Technology (GIST), 123 Cheomdangwagi-ro, Buk-gu, Gwangju 61005, Korea; min90821@gm.gist.ac.kr (C.L.); yesol7964@gist.ac.kr (Y.K.); dhowon16@gist.ac.kr (D.-H.K.)

**Keywords:** reverse osmosis, hybrid desalination, boron removal

## Abstract

The wastewater–seawater (WW-SW) integrated reverse osmosis (RO) process has gained much attention in and out of academia due to its energy saving capability, economic benefits, and sustainability. The other advantage of this process is to reduce boron concentration in the RO permeate that can exclude the post-treatment process. However, there are multiple design constraints regarding boron removal that restrict process design in the WW-SW integrated system. In this study, uncertainties in design factors of the WW-SW integrated system in consideration of boron removal have been explored. In comprehensive consideration of the blending ratio of between WW and SW, regulatory water quality standard, specific energy consumption (SEC), specific water cost, and RO recovery rate, a range of 15,000~20,000 mg/L feed turned out to be the most appropriate. Furthermore, boron rejection tests with SWRO (seawater reverse osmosis) and BWRO (brackish water reverse osmosis) membranes under actual WW-SW integration found a critical reduction in boron rejection at less than 20 bar of operating pressure. These findings emphasize the importance of caution in the use of BWRO membranes in the WW-SW integrated RO system.

## 1. Introduction

Boron is a common element in the environment and the 10th most abundant ionic species in the ocean [1]. Boron concentration in the ocean varies depending on the site and season, but the global average of boron concentration has been widely known as approximately 4.8 mg/L [2]; yet, it can range from 7 up to 13 mg/L in certain regions such as the Arabian Gulf and the Mediterranean Sea [3]. Due to the toxicity of boron for human health and agriculture, boron concentration is commonly regulated around the world especially in nations where seawater desalination is employed for obtaining drinking water or irrigation water. For example, an acceptable level of boron for potable water in the guideline of the World Health Organization (WHO) was originally set at below 0.5 mg/L but was modified to 2.4 mg/L in 2012, while European Union (EU) and Spanish legislation constantly maintain it as 1.0 mg/L [4,5,6]. This regulation on boron concentration in the final product of seawater desalination acts as a design constraint in the process design.

Boron predominantly exists as a form of boric acid in the typical ocean pH (7–8) [7]. This boric acid in seawater is especially difficult to remove using membrane technology due to its neutral charge characteristic and small hydrated radius compared to other elements [8]. In the equilibrium reaction of Equation (1), boric acid exponentially decreases and transforms into borate ions with increasing pH. Since borate ions have a larger fully hydrated radius and negative charge, the rejection rate for this dissociated form of boron significantly increases both by size exclusion and charge repulsion of the negatively charged membrane [9]. In the case of a typical commercial seqater reverse osmosis (SWRO) membrane, boron rejection by the SWRO membrane can elevate up to about 99% and for brackish water reverse osmosis (BWRO) membranes to 93%, while at pH 11, these values increase to to 99.5% for SWRO and 99.0% for BWRO membranes [9].

(1) $$B{\left(\mathrm{OH}\right)}_{3}+H_{2}O\;⇋B{\left(\mathrm{OH}\right)}_{4}{}^{-}+H^{+}=,\;pK_{a}=9.2\;\mathrm{at}\;\mathrm{pH}\;7\;\mathrm{and}\;25\;{}^{\circ}C$$

Due to such efficient boron removal in high-pH conditions, a multiple-pass RO configuration with pH adjustment for boron rejection is typically employed in the seawater reverse osmosis (SWRO) [10], which imposes an additional cost burden in water production cost. Such post-treatment processes increase capital and operating costs and resulted an increase of water production cost between 0.04 and 0.1 $/m^3^ [11,12].

The hybrid desalination process integrating SWRO with wastewater reuse has attracted large research attention due to the potential reduction of energy consumption and brine disposal [13]. In the hybrid scheme, impaired water such as municipal or industrial wastewater is used to dilute seawater through osmotic dilution, such as forward osmosis (FO) and pressure-assisted forward osmosis (PAFO), or directly mixed after ultrafiltration (UF) pretreatment [14,15]. Up to date, there is no literature reporting the practical implementation of the SWRO using directly mixed wastewater (WW) and seawater (SW) as a feed. This is most likely due to high fouling potential, especially for biofouling, from insufficient removal of carbon species in treated wastewater [16]. One of the benefits of this hybrid process is that post-treatment loading for boron removal can be reduced due to dilution of the initial boron concentration in the seawater. In this regard, a recent study [17] evaluated boron rejection of RO and FO membranes using blended WW-SW water. Although recent studies suggested that an additional boron removal process is not required in the hybrid process [16,17], it is still unclear whether this boron treatment can be practically exempted under any circumstances of the hybrid process. Technically, the requirement of additional boron treatment is determined by practical considerations in process design. For example, municipal wastewater generally contains up to 0.6 mg/L [18], and considering marginal boron rejection of the FO membrane [4,19], boron concentration in wastewater can significantly affect the dilution of boron in seawater. Furthermore, design factors in the RO process, such as the selection of the RO membrane and element configuration [5] or operating feed pressure [10,20], dictate boron rejection rate in the RO process. Especially, operating feed pressure in the hybrid process is most likely to be lower than that in conventional SWRO process to benefit from a lower concentration of diluted seawater. Boron rejection in the RO process also can be dictated by characteristics of the feed water such as pH, constituent ions, and operating conditions such as water flux, cross-flow velocity, and temperature [4,10,19,20,21]. Such considerations can have a significant influence on the process design of the hybrid scheme in the aspect of satisfying the regulation of boron concentration in the final water product.

The concept of using WW-SW blended feed water in the RO system has long been studied in academia; however, there is a clear need to investigate and validate before applying this idea to large-scale plants. In particular, the design consideration of boron removal is still left unclear. The objective of the current study is to evaluate practical considerations in the wastewater–seawater (WW-SW) integrated desalination for boron removal and the resultant design process and economics of the hybrid process. This study will provide a practical guideline of the hybrid process in terms of boron removal in comprehensive consideration of design factors such as the blending ratio between WW-SW, the regulatory standard for boron concentration, specific energy consumption (SEC), specific water cost, and RO recovery rate. Moreover, two types of RO membranes (SWRO, BWRO) were evaluated under a range of operating pressure for practical feasibility under actual WW and SW conditions. This study is expected to shed new light on the consideration of boron removal in the design of the WW-SW integrated RO system.

## 2. Materials and Methods

### 2.1. Membrane Transport Equations

The water flux ($J_{v}$) and solute flux ($J_{s}$) through the membrane in reverse osmosis can be described by:

(2) $$J_{v}=A\left(\Delta P-\sigma \Delta \pi \right)$$

(3) $$J_{s}=B\left(C_{m}-C_{p}\right)$$

where *A* is the water permeability, *B* is solute permeability, σ is the reflection coefficient, ΔP is the pressure difference across the membrane, Δπ is the osmotic pressure difference across the membrane, and $C_{m}$ and $C_{p}$ are solute concentration on the membrane surface in feed and permeate side, respectively.

The solute concentration at a membrane surface can be estimated from that in a bulk by:

(4) $$\frac{C_{m}-C_{p}}{C_{B}-C_{p}}=\exp\left(\frac{J_{v}}{k}\right)$$

where *k* is the mass transfer coefficient of solute and $C_{B}$ is the bulk solute concentration.

### 2.2. Membrane and Spacers

Commercial thin-film nanocomposite (TFN) SWRO (LG 400 GR) and BWRO (BW 400 R) membranes were provided from LG Chem (Seoul, Korea). The membranes were obtained as flat sheets about 1 × 1.5 m^2^ and dry state in sealed bags. Both TFN membranes consisted of a selective polyamide active layer formed by interfacial polymerization on top of a polysulfone porous substrate. Before experimental use, both membranes were soaked in deionized (DI) water and stored at 4 °C.

### 2.3. Feed and Draw Solutions

Secondary wastewater effluent was collected from a wastewater treatment plant (Yeosu Wastewater Treatment Plant, Yeosu, Korea), and seawater was collected from the South Sea (34°43′58.9″ N 127°40′52.7″ E). The collected wastewater and seawater samples were blended to produce mixed solutions with 15,000 and 20,000 mg/L of total dissolved solids (TDS) concentration. Produced solutions were stored at 4 °C and used in the experiments within 3 days. 

Each water quality parameter of feed and draw solutions was measured before and after pretreatment (Table 1 and Table 2). Total organic carbon (TOC) was measured using the TOC analyzer (TOC-L, Shimadzu, Kyoto, Japan). Suspended solids (SS) and silt density index (SDI) were determined by following the procedure of standard methods (ASTM, 2014). Ion chromatography (ICS-3000, Dionex, California, CA, USA) was used to analyze cation contents in each solution. 

### 2.4. Lab-Scale RO System Setup

A reverse osmosis membrane performance experiment was carried out using a flat sheet membrane filtration cell (SEPA CF II, GE Osmonics, Minnesota, MN, USA). Briefly, 15,000 and 20,000 ppm mixed feed solutions were prepared in a 10 L stainless tank. Feed solution temperature was kept by adjusting the chiller temperature set point 25 ± 0.5 °C. Operation pressure was controlled with a high-pressure pump (2SF29SELL CAT Pumps; Minneapolis, MI, USA) and a LEO2 digital manometer (Keller AG, WInterhur, Swltzerland) from 20 to 50 bar. The scale connected with a computer was used for measuring permeate water flux. Every feed and permeate solution was sampled for assessing boron concentration and TDS. For the seawater feed solution, the experiment was conducted using an SWRO at 50 bar and afterward using a BWRO at 15 bar. Each test run was preceded by 2 h of membrane compaction under 50 bar.

### 2.5. Boron Concentration Measurement

To measure boron concentration in the samples, a TNT 877 (Boron) vial kit (HACH, Loveland, CO, USA), which is applied for wastewater, seawater, drinking water, surface water, and produced water, was used. The measurement procedure was carried out according to the kit’s manual, and the procedure is as follows:

(1) Carefully pipet 1.0 mL of solution A into the test vial.
(2) Carefully pipet into the same vial: 2.5 mL of sample.
(3) Close the vial, swirl the contents, and invert several times until the lyophilizate has dissolved completely.
(4) After 40 min, thoroughly clean the outside of the vial and evaluate.
(5) Insert the vial into the cell holder of the spectrophotometer.

To read the fluorescence from the sample, a DR 2800 spectrophotometer (HACH, Loveland, CO, USA) was used. Since the boron measurement range of the TNT 877 kit is 0.0–2.5 mg/L, the samples were diluted to adjust the appropriate boron concentration range before the measurements.

### 2.6. Simulation Method

Permeate boron concentration, boron rejection, TDS rejection, SEC, and specific water cost in the wastewater–seawater integrated two-stage reverse osmosis process with a final product rate of 100,000 m^3^/d was estimated using Q+ Projection Software (LG Chem Water Solution, Seoul, Korea). Target RO recoveries varied from 40% to 80% in the simulation. LG SW 400 ES, SW 400 R, and BW 400 ES elements were selected as model SWRO elements with high flux, high rejection, and BWRO membrane, respectively, with varying input concentrations from 10,000 to 45,000 mg/L NaCl to mimic a wide range of concentrations of the diluted draw streams. 

## 3. Results and Discussion

### 3.1. Considerations of TDS and Boron Concentration in WW-SW Blending

**Figure 1:**
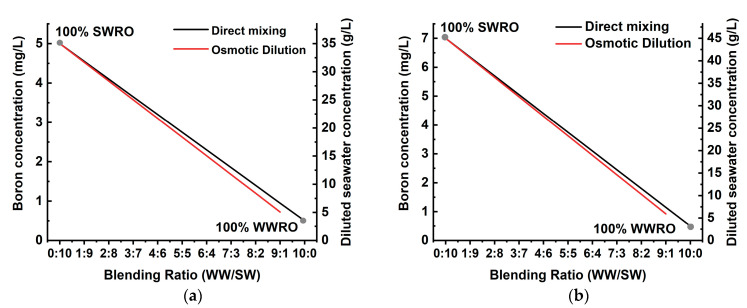
Variation of boron concentration coupled with TDS concentration of seawater by blending ratio of treated wastewater and seawater in (**a**) Arabian Gulf and (**b**) the average of seawater concentration.

**Figure 2:**
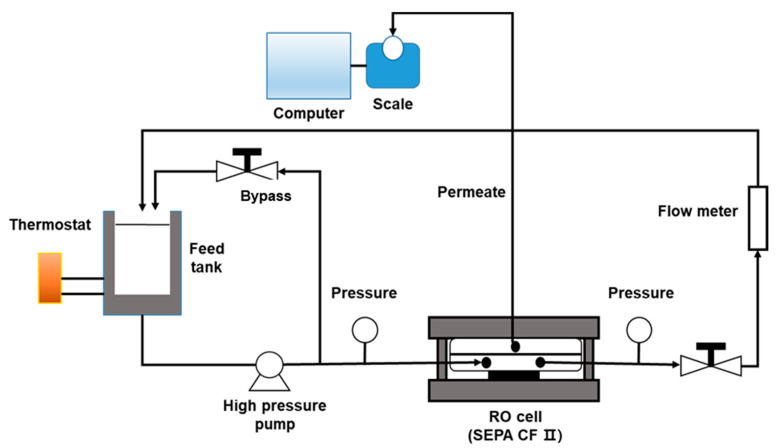
Schematic diagram of a lab-scale reverse osmosis (RO) system.

In the mixed solution of treated wastewater (WW) and seawater (SW), the initial boron concentration in SW decreases with dilution by blending WW that contains relatively marginal boron concentration (0.5 mg/L) compared to pure SW. As aforementioned, the global average of boron concentration in seawater is 4.8 mg/L, but it can reach up to more than 7 mg/L in the Arabian Gulf where more than half of desalination plants of the world were located and being operated [22]. For process simulation in the current study, the initial concentration of TDS and boron in SW were assumed to be 35,000 and 5 mg/L for the case of average seawater, and 45,000 and 7 mg/L for the Arabian Gulf. The TDS and boron concentration of WW were assumed to be 1000 and 0.5 mg/L. Figure 1 shows the variation of boron concentration coupled with corresponding TDS concentration as a function of the blending volume ratio of WW and SW in the case of direct mixing and osmotic dilution. In osmotic dilution, the resultant boron concentration is slightly lower than that in direct mixing due to the rejection of boron in treated wastewater by the FO membrane. TDS and boron concentration in osmotic dilution were computed using the separation performance of a commercial forward osmosis membrane as reported in the literature (water flux: 20.6 L/m^2^/h, reverse solute flux: 6.3 g/m^2^/h, reverse boron flux: 0.005 mg/m^2^/s, boron rejection: 50%) [17]. As shown in Figure 2, a higher volume proportion of treated wastewater is beneficial to reduce boron concentration in the diluted seawater, mitigating the burden of boron treatment in the subsequent SWRO process. 

However, it should be noted that the blending ratio of WW to SW can be subject to either available capacity of treated wastewater in direct mixing or process design of FO or PAFO (i.e., operating condition and configuration of membrane element). The osmotic dilution process can provide a higher level of pretreatment to the treated wastewater by removing most of the dissolved and colloidal carbon species. For this reason, the FO–RO hybrid process has been more actively studied in the form of pilot and feasibility studies. Existing pilot studies on FO–RO hybrids are listed in Table 3. Due to dilution efficiency [23] or the overall economics [13] of the hybrid process, the range of diluted seawater concentration generally falls into approximately 25,000~10,000 mg/L in average seawater concentration (35,000 mg/L) and 20,000 to 30,000 mg/L in the Arabian Gulf (45,000 mg/L), corresponding 3.16~1.63 and 4.34~3.01 mg/L of boron concentration, respectively. This boron concentration of mixed solution determines the permeate boron concentration with process design of the subsequent SWRO. Thus, the blending ratio of WW and SW can be constrained with SWRO design (e.g., staging design and RO membrane selection) to satisfy the target boron concentration in the final product. In this aspect, the optimal blending ratio between treated wastewater (WW) and seawater (SW) will be drawn in consideration of system performance parameters such as specific energy consumption (SEC) and specific water cost.

### 3.2. Process Simulation of WW-SW Integrated SWRO

To examine the influence of blending ratio in an WW-SW integrated SWRO on system performance, the variation of each parameter was drawn as a function of feed TDS concentration, RO recovery rate, and type of RO membranes. A range of feed TDS concentrations (10,000, 15,000, 20,000, 30,000, 35,000, and 45,000 mg/L) and RO recovery rates (40~80%) were selected by postulating practical range based on existing pilot studies (Table 1). Three different RO membranes—high flux SWRO, high rejection SWRO, and BWRO (brackish water reverse osmosis) membrane—were chosen to evaluate the influence of membrane choice. Although the BWRO membrane was originally developed to treat brackish water (under 10,000 mg/L), some studies suggest that the BWRO membrane can be employed to improve the economics of the FO–RO hybrid system [13,27].

#### 3.2.1. Permeate Boron Concentration and Rejection Rate

**Figure 3:**
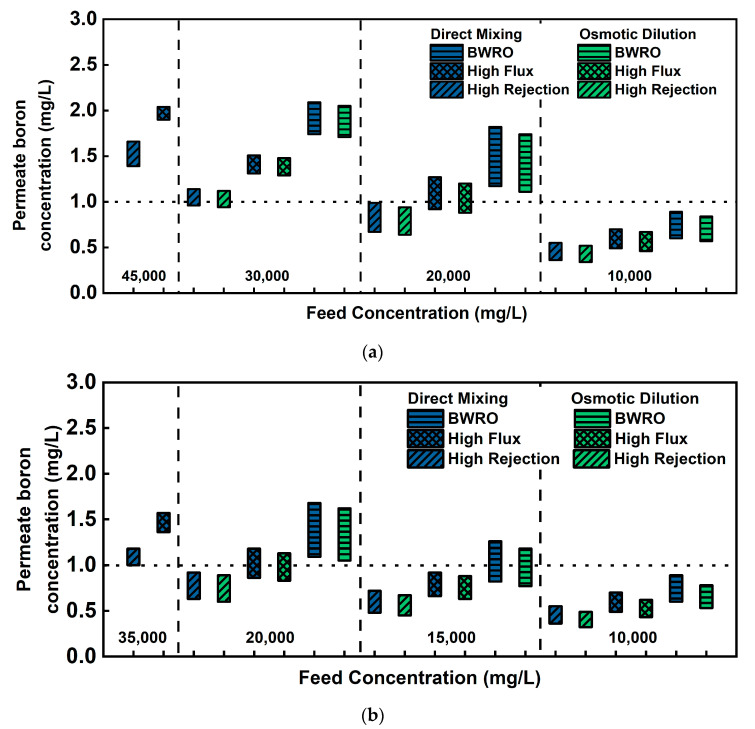
Variation of permeate boron concentration in the final product by process configuration, feed concentration, and membrane types in (**a**) Arabian Gulf seawater and (**b**) average seawater.

Generally, boron rejection is much lower than TDS rejection due to its unique aquatic chemistry [8,20]. As aforementioned, 1.0 mg/L of boron concentration is the maximum level, which is the most commonly accepted over the world although some seawater desalination plants set limits as low as 0.5 mg/L for water for agriculture use [4]. Figure 3 depicts how the permeate boron concentration varies depending on its process configuration (direct mixing of feed or osmotic dilution), feed concentration, and membrane types in two postulated feed water conditions (Arabian Gulf and average seawater). Each end of the floating bar indicates the minimum and maximum level of permeate boron concentration by RO recovery rate (40~80%). In the case of Arabian Gulf seawater, minor dilution (30,000 mg/L) does not satisfy the regulatory standard of boron concentration. Feed water needs to be diluted to at least 20,000 mg/L to partially meet regulatory standards depending on membrane types and to 10,000 mg/L for all types of the membrane to comply with the standard. In the average seawater condition, 20,000 mg/L partially satisfies the standard and 10,000 mg/L is the concentration level where boron concentration less than the standard is assured regardless of membrane types as in the case of Arabian Gulf seawater. These results suggest that feed seawater needs to be diluted to 10,000 mg/L to sufficiently meet the regulatory standard for all types of membranes in both Arabian Gulf and average seawater. However, 10,000 mg/L requires approximately an 8:2 ratio of WW/SW blending for both seawater conditions, which is not quite feasible considering the fact that WW flow rate is limited in most cases. In this respect, 20,000 mg/L of diluted feed concentration requires reasonable blending ratio (approximately 5:5) and satisfies the standard with limited selection of membrane types. Difference in permeate boron concentration by process configuration was insignificant to be considered a decisive factor in process design, but it can be handy to fill the small concentration gap or delay the membrane replacement or cleaning to recover boron rejection rate.

**Figure 4:**
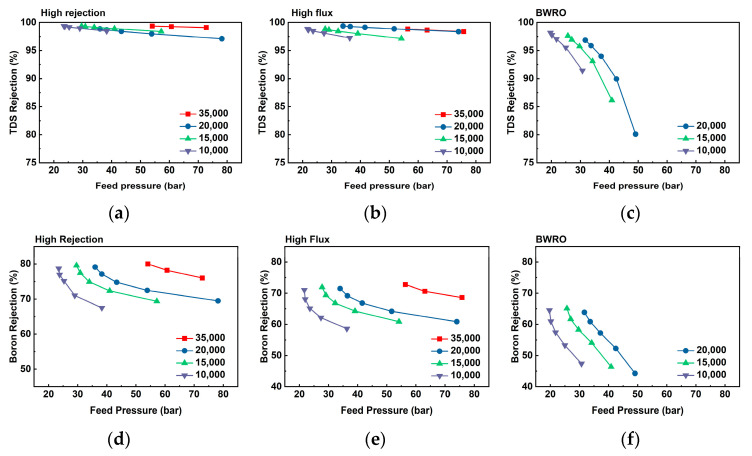
Boron rejection and TDS rejection by feed pressure by using high rejection membrane (**a**,**d**), high flux membrane (**b**,**e**), and braish water reverse osmosis (BWRO) membrane (**c**,**f**).

A range of recovery rate (40~80%) used in a set of simulations induces a difference in feed pressure that causes the variation of TDS and boron rejection rate. In Figure 4, varied TDS and boron rejection were plotted as a function of feed pressure of different recovery rates. BWRO showed a drastic decrement in both TDS and boron rejection as feed pressure increases. Furthermore, TDS and boron rejection tend to decrease more dramatically under a lower level of feed concentrations. This trend of TDS and boron rejection can be associated with concentration polarization (CP). The presence of high TDS concentration in SW can result in CP, which is an undesirable phenomenon as it induces an enhanced solute concentration on the membrane surface. The most deleterious CP effect lies in deteriorating the risk of precipitation of a sparingly soluble salt, by enhancing its concentration on the membrane surface [28,29]. This elevated level of TDS and boron concentration reduces TDS and boron rejection. Since higher feed pressure induces a higher level of CP and saturated concentration on the membrane surface, TDS and boron rejection decrease accordingly.

#### 3.2.2. Specific Energy Consumption (SEC) and Specific Water Cost

**Figure 5:**
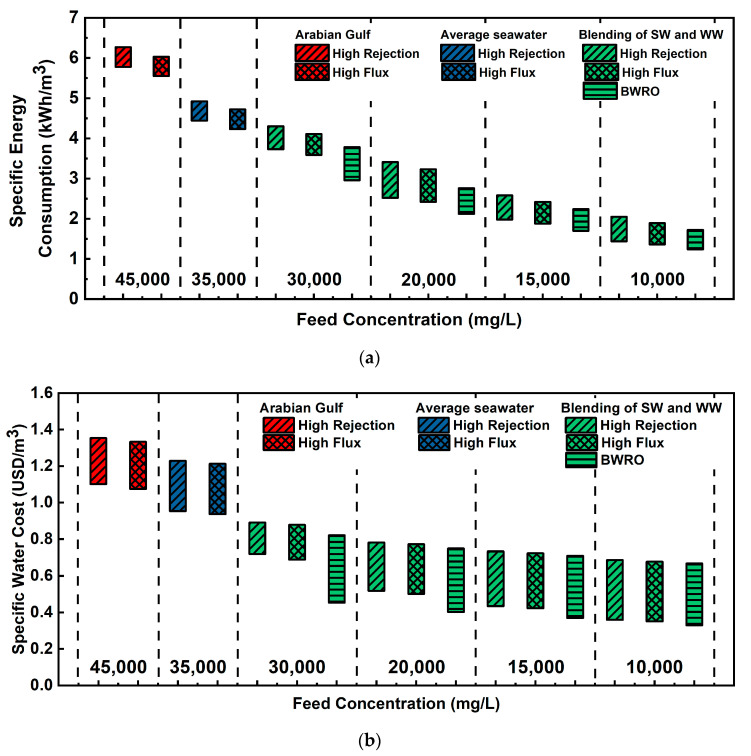
(**a**) Specific energy consumption (SEC) variation by feed concentration, recovery rate, and RO membrane types. (**b**) Water cost variation by feed concentration, recovery rate, and RO membrane types.

**Figure 6:**
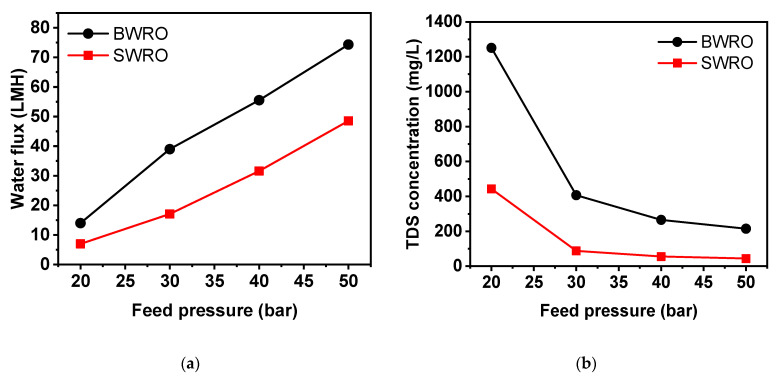

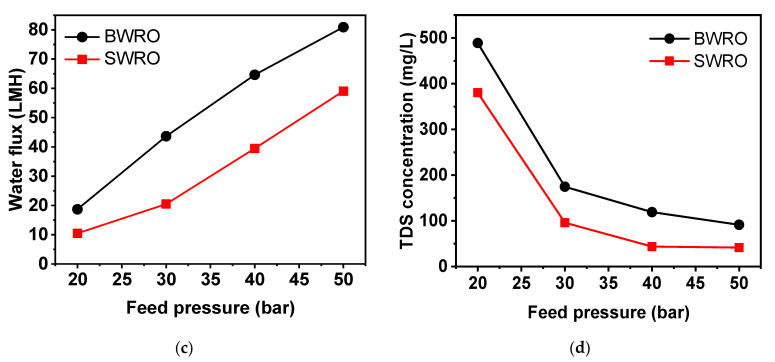
Variation of water flux and permeate TDS concentration by applied hydraulic pressure and membrane types (seawater reverse osmosis (SWRO) and BWRO) in feed concentration of 20,000 mg/L (**a**,**b**) and 15,000 mg/L (**c**,**d**).

Specific energy consumption (SEC) is the unit energy consumption per cubic meter of final product waters of the desalination processes. Since energy cost is the parameter with the greatest effect on the economics of an RO desalination plant [30], it should be considered in plant design with other design factors. Figure 5 shows SEC variation by feed TDS concentration and membrane type. Each end of the floating bar depicts minimum and maximum SEC depending on the RO recovery rate (40~80%). As feed concentration decreases by feed blending, SEC drops down to 1.24 kWh/m^3^ from undiluted feed concentration (5.55~6.27 and 4.23~4.93 kWh/m^3^ for Arabian Gulf and average seawater concentration, respectively). Although there was a distinct difference in SEC by membrane types over 20,000 mg/L, SEC benefits by using the BWRO membrane gradually decreased from 20,000 mg/L (0.4~0.65 kWh/m^3^) toward 10,000 mg/L (less than 0.3 kWh/m^3^). Considering that the BWRO membrane cannot be used as it does not meet the boron regulatory standard of more than 20,000 mg/L, the merit of using the BWRO membrane under 20,000 mg/L can be not significant as known in the other literature. These insignificant benefits of using the BWRO membrane can also be found in the specific water cost analysis (Figure 6). Although the BWRO membrane demonstrated a certain extent of water cost reduction, there is no meaningful improvement below 20,000 mg/L. Additionally, producing feed water with a concentration lower than 20,000 mg/L requires an impractical blending ratio (i.e., higher than 7:3 (WW:SW)). In other words, using the BWRO membrane for a WW-SW integrated RO system is hardly feasible or applicable in very limited circumstances in consideration of the SEC and specific water cost. 

### 3.3. Lab-Scale Evaluation of Boron Removal

To examine the practical performance of the RO membrane under WW and SW conditions, lab-scale RO experiments were conducted using SWRO and BWRO membranes. Actual seawater and secondary wastewater influents were used for the performance evaluation. Water quality parameters of feed waters are provided in Table 1 and Table 2. As discussed above, the optimal range of diluted feed concentration drawn from the simulations is 15,000~20,000 mg/L. Thus, two feed solutions with TDS concentration 15,000 and 20,000 mg/L were prepared by diluting SW with WW to the designated concentrations. SW and WW were pretreated with a 0.45 um UF membrane filter before mixing. Since the range of RO recovery rates used in this study induces a variation of feed pressure down to approximately 20 bar, four pressure points from 20 to 50 bar were selected to evaluate RO performance.

**Figure 7:**
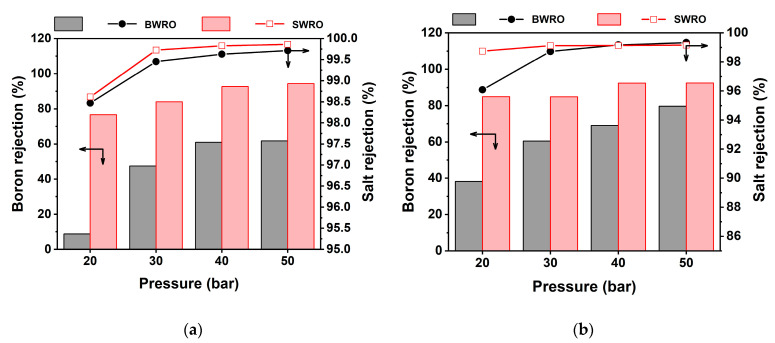
Boron and salt rejection by applied hydraulic pressure and membrane types (SWRO and BWRO) in feed concentration of (**a**) 20,000 mg/L and (**b**) 15,000 mg/L.

Figure 7 depicts the behavior of flux and TDS rejection under a range of feed pressure with SWRO and BWRO membranes. In a given range of feed pressure, water flux showed a positive linear correlation to feed pressure for both membranes. Water flux of SWRO is constantly approximately 20 LMH higher compared to that of BWRO (from 30 to 50 bar) but showed much smaller difference (less than 10 LMH) at 20 bar. Furthermore, permeate TDS concentration at 20 bar was a lot higher than that above 30 bar, and TDS concentration exponentially decreases with increasing feed pressure to 50 bar. This irregularity in RO membrane performance at low-pressure conditions (less than 20 bar) can be attributed to the structural change of the RO membrane under hydraulic pressure that is lower than originally designed operating conditions. More specifically, apparent RO membrane performance (both water permeability and rejection) is assured by densified membrane structure (i.e., membrane compaction) under the designed hydraulic pressure for that specific membrane. The membrane tends to return to the loosened state under lower hydraulic pressure due to the elasticity of the polymers, and this makes a difference in membrane performance as shown in Figure 6. Given that the membrane went through membrane compaction at 50 bar for each test run, both RO membranes were quick to bounce back to a loosened state. This discrepancy in membrane performance by the different extent of membrane compaction is known to be more dominant in solute permeability than in water permeability [14,31]. Furthermore, this propensity is more predominant in higher concentrations (20,000 mg/L) than in the lower feed concentration (15,000 mg/L). As shown in Figure 7, the rejection of boron and TDS decreases dramatically with decreasing feed pressure, especially at 20 bar. While TDS rejection keeps a relatively higher level, boron rejection turned out to be highly sensitive to applied hydraulic pressure as it decreases down to less than 10% at 20 bar. Although several studies [17,20,32] demonstrated the improvement in the relation between high hydraulic pressure to boron rejection at a high range of pressure (more than 40 bar), this study is the first to report a drastic boron rejection decrease in the low-pressure range. This result is crucial as there is no consideration of boron rejection change under different feed pressures in conventional modeling or simulations. Since several studies suggest low operating pressure in WW-SW integrated RO system [13,33,34], this boron rejection drop under low operating pressure must be considered to meet regulatory water quality standards for permeate boron concentration in the final product. 

### 3.4. Practical Considerations of Boron Removal in WW-SW Integrated RO System

The minimum blending ratio of WW and SW to meet boron concentration standards is the most important design factor for the WW-SW integrated RO system as it determines the maximum allowable plant capacity, RO operating conditions, and fouling propensity based on many other design factors. As mentioned earlier, the target diluted concentration of blended feed water has to be below 20,000 mg/L (i.e., equivalent to a 5:5 blending ratio) to satisfy the standard for permeate boron concentration. However, several factors can reduce boron rejection that changes the minimum blending ratio. First, a high RO recovery rate can reduce boron rejection as high water flux deteriorates CP and eventually reduces boron rejection (Figure 4). High RO recovery not only reduces boron rejection but also increases fouling and scaling potential; thus, a high level of pretreatment [35] is required to not constrain the RO process design. Temperature and salinity of the feed water also can reduce boron rejection since boric acid is more easily dissociated under high feed salinity and temperature [2]. This can be specifically critical in the Arabian Gulf where the seawater temperature rises to 40 °C on top of the high feed boron concentration. As found in the lab-scale experiments (Figure 7), significantly low boron rejection under low feed pressure can be a critical design constraint, especially with BWRO membranes. The above practical aspects have to be carefully considered to maintain stable boron permeate concentration and maximize the economics of the plant simultaneously.

## 4. Conclusions

Dilution of seawater with other water sources is one of the efficient ways to reduce permeate boron concentration that improves the overall economics of the WW-SW integrated RO system by excluding additional post-treatment processes. However, there are many practical aspects to constrain the system design in consideration of boron removal. This study examines possible design constraints for boron removal by using simulation and experimental methods. Under average and Middle Eastern seawater conditions, 15,000 to 20,000 mg/L turned out to be appropriate in light of a practical mixing ratio and target boron concentration in the final product. However, high RO recovery, feed salinity, and temperature can potentially reduce boron rejection and affect the RO process design. Although the use of the BWRO membrane has been suggested by many studies, the low rejection capacity of BWRO under a given range of feed concentration was not sufficient to meet target standards. Performance evaluation of both SWRO and BWRO membranes under actual SW and WW conditions revealed critically low boron rejection under low operating pressure (less than 20 bar) for both membranes. Since this experimental finding is not accounted for in the conventional simulation, the practical feasibility of such design factors needs to be reconsidered for design and implementation in the WW-SW integrated RO system.

## Figures and Tables

**Table 1:** Characteristics of seawater and secondary wastewater effluent.

Water Quality Parameter	Secondary Wastewater Effluent		Seawater	
	Raw	Pretreated	Raw	Pretreated
**TDS (mg/L)**	3148±142		33,597±154	
**Turbidity (NTU)**	2.1±3.7	0.5±0.23	0.6±0.32	0.2±0.09
**TOC (mg/L)**	6.2±0.81	5.1±0.8	1.7±0.01	1.6±0.03
**SS (mg/L)**	6±2.18	3±0.5	2.4±0.6	0.8±0.7

TDS, total dissolved solids; TOC, total organic carbon; SS, suspended solids.

**Table 2:** Major constituent ions of seawater and secondary wastewater effluent (mg/L).

Ion Composition	Secondary Wastewater Effluent	Seawater
**K^+^**	16.7	392
**Na^+^**	195	9242
**Ca^2+^**	31.8	427
**Mg^2+^**	22	1350
**F^−^**	0.096	0.825
**NO^3−^**	25.5	0.810
**SO_4_^2−^**	114	2954
**Cl^−^**	354	21,392
**Br^−^**	0.819	66.1

**Table 3:** List of diluted concentration in pilot studies of the wastewater–seawater (WW-SW) integrated RO process with operating condition and membrane types.

Process	Element Type	Operating Condition	Diluted Concentration	Reference
FO	HTI CTA FO 4040	Initial Draw Concentration: 35,000 mg/L Feed flow rate: 6 m^3^/day Draw flow rate: 6 m^3^/day Recirculation	Approximately 22,000~10,000 mg/L	[24]
FO/PAFO	Toray CSM FO-8040	Initial Draw Concentration: 35,000 mg/L Feed flow rate: 30~60 LPM Draw flow rate: 2~6 LPM Hydraulic pressure difference: 0, 4 bar Serial Configuration	14,000~9300 mg/L	[25]
PAFO	Toray CSM FO-8040	Initial Draw Concentration: 35,000 mg/L Feed flow rate: 50~70 LPM Draw flow rate: 5~7 LPM Hydraulic pressure difference: 0~4 bar Serial Configuration	19,656~11,118 mg/L	[13]
PAFO	Toray CSM FO-8040	Initial Draw Concentration: 32,258 mg/L Feed flow rate: 10~70 LPM Draw flow rate: 2.5~7.5 LPM Hydraulic pressure difference: 0~4 bar Serial Configuration	23,111~7500 mg/L	[23]
FO	Toray CSM FO-4040 Porifera PFO-100	Initial Draw Concentration: 45,000 and 35,000 mg/L Feed flow rate: 3.3~6.6 LPM (Toray) 15~40 LPM (Porifera) Draw flow rate: 0.35~0.55 LPM (Toray) 15~40 LPM (Porifera) Recirculation	(45,000 mg/L) -Toray 32,434~19,190 mg/L -Porifera 40,245~26,490 mg/L (35,000 mg/L) -Toray 31,744 ~22,519 mg/L -Porifera 27,403~17,906 mg/L	[26]

FO, forward osmosis; PAFO, pressure-assisted forward osmosis; LPM, Liter per minute.
